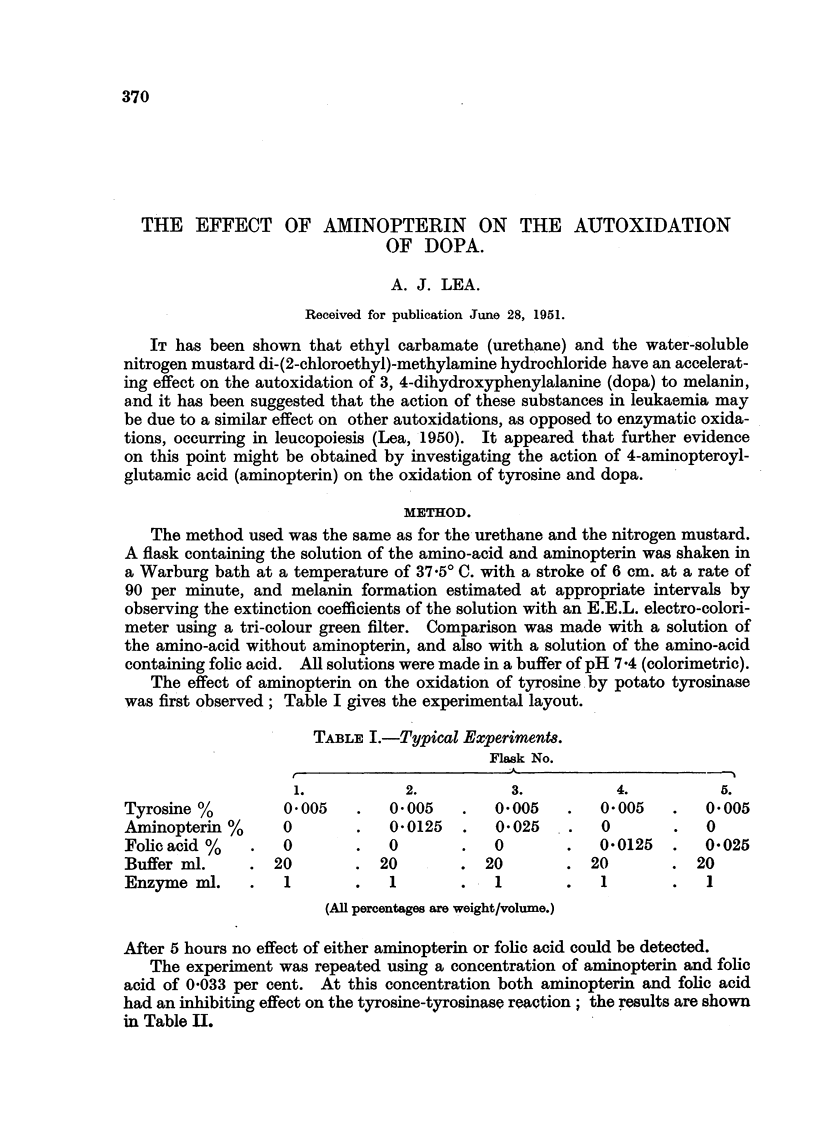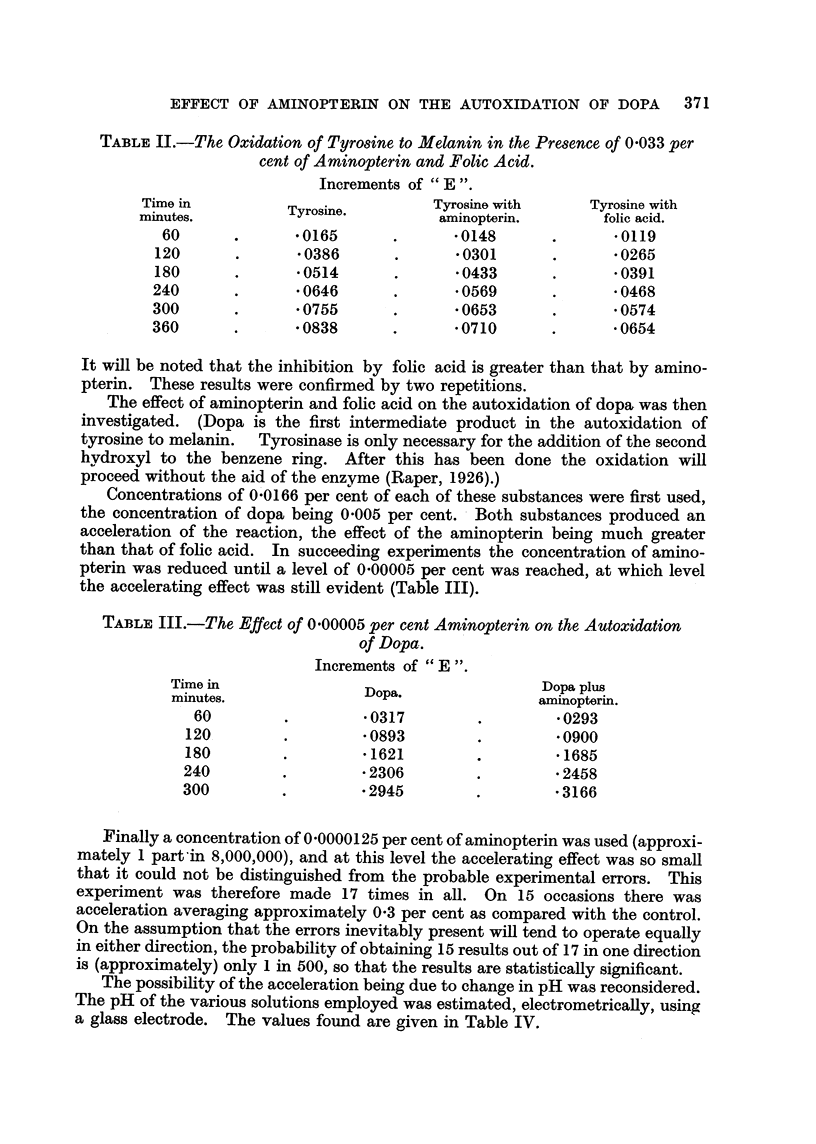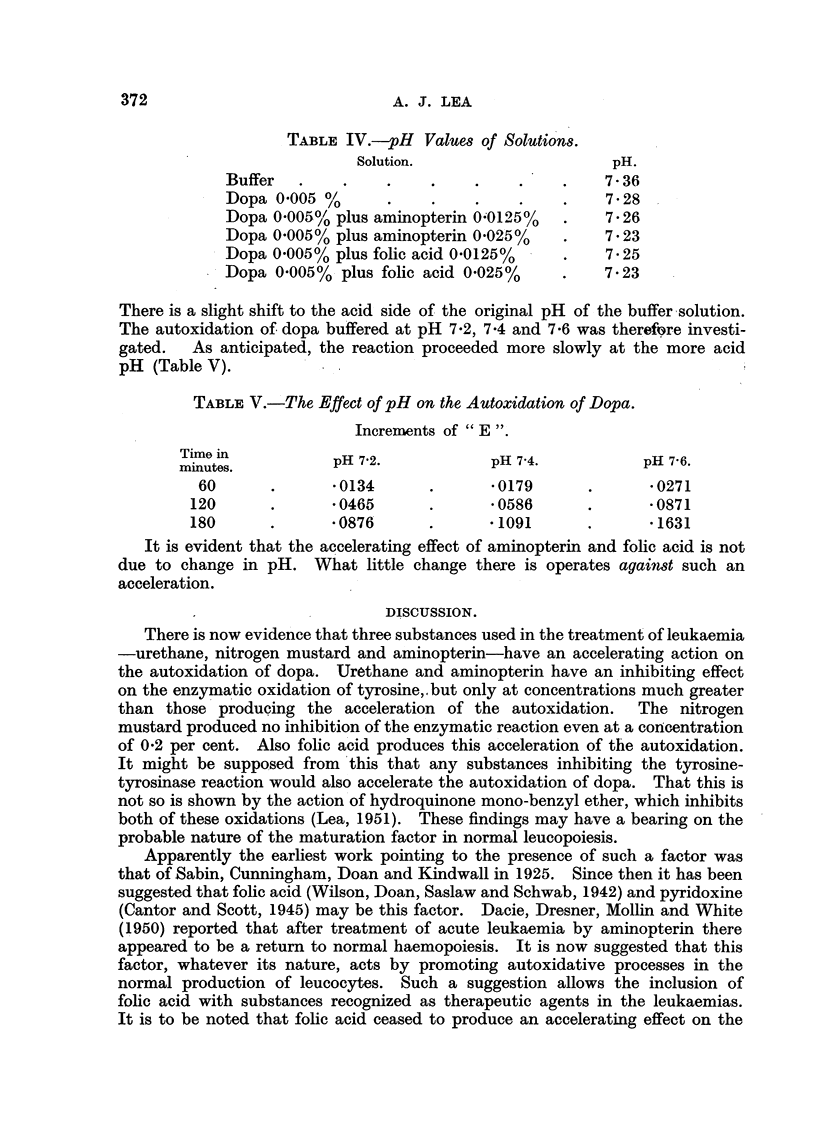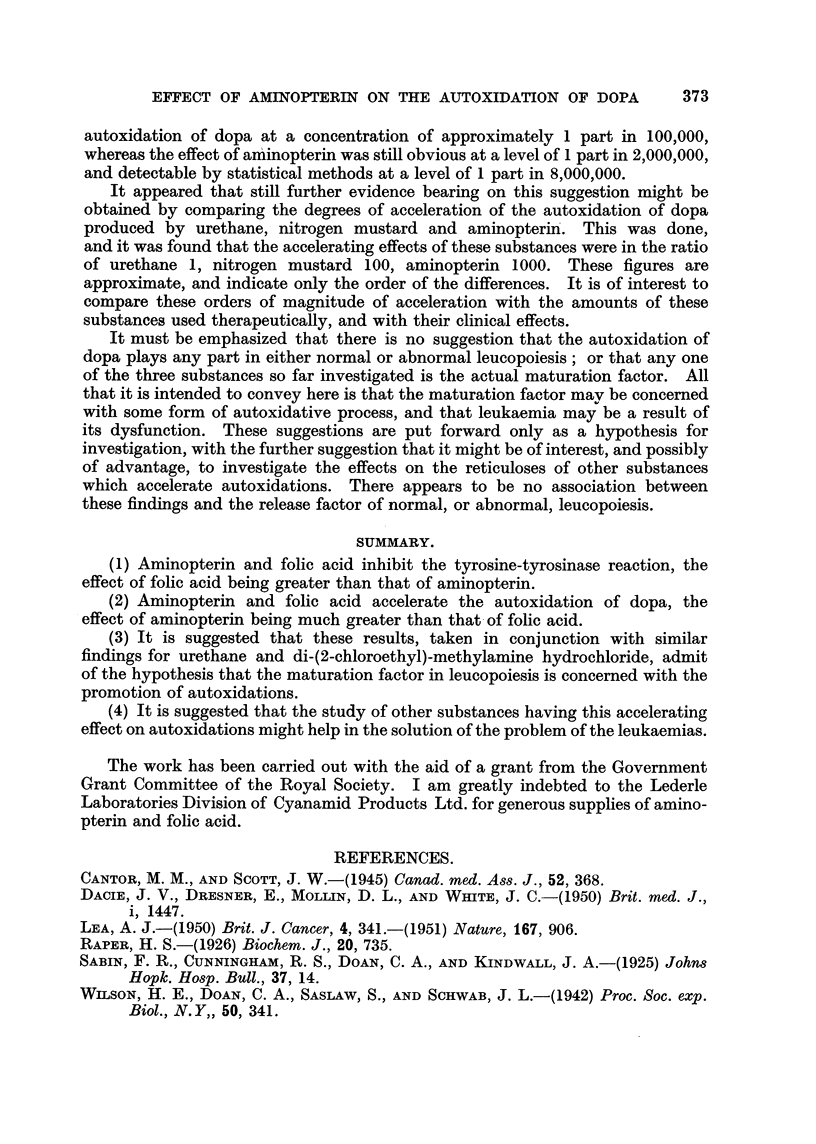# The Effect of Aminopterin on the Autoxidation of Dopa

**DOI:** 10.1038/bjc.1951.40

**Published:** 1951-09

**Authors:** A. J. Lea


### THE EFFECT OF AMINOPTERIN ON THE AUTOXIDATION OF DOPA.

A. J. LEA.

Received for publication June 28, 1951.

It has been shown that ethyl carbamate (urethane) and the water-soluble nitrogen mustard di-(2-chloroethyl)-methylamine hydrochloride have an accelerating effect on the autoxidation of 3, 4-dihydroxyphenylalanine (dopa) to melanin, and it has been suggested that the action of these substances in leukaemia may be due to a similar effect on other autoxidations, as opposed to enzymatic oxidations, occurring in leucopoiesis (Lea, 1950). It appeared that further evidence on this point might be obtained by investigating the action of 4-aminopteroylglutamic acid (aminopterin) on the oxidation of tyrosine and dopa.

### METHOD.

The method used was the same as for the urethane and the nitrogen mustard. A flask containing the solution of the amino-acid and aminopterin was shaken in a Warburg bath at a temperature of 37·5° C. with a stroke of 6 cm. at a rate of 90 per minute, and melanin formation estimated at appropriate intervals by observing the extinction coefficients of the solution with an E.E.L. electro-colorimeter using a tri-colour green filter. Comparison was made with a solution of the amino-acid without aminopterin, and also with a solution of the amino-acid containing folic acid. All solutions were made in a buffer of pH 7·4 (colorimetric).

The effect of aminopterin on the oxidation of tyrosine by potato tyrosinase was first observed ; Table I gives the experimental layout.

TABLE I.—*Typical Experiments.*

| | Flask No. 1. | 2. | 3. | 4. | 5. |
|---|---|---|---|---|---|
| Tyrosine % | 0·005 | 0·005 | 0·005 | 0·005 | 0·005 |
| Aminopterin % | 0 | 0·0125 | 0·025 | 0 | 0 |
| Folic acid % | 0 | 0 | 0 | 0·0125 | 0·025 |
| Buffer ml. | 20 | 20 | 20 | 20 | 20 |
| Enzyme ml. | 1 | 1 | 1 | 1 | 1 |

(All percentages are weight/volume.)

After 5 hours no effect of either aminopterin or folic acid could be detected.

The experiment was repeated using a concentration of aminopterin and folic acid of 0·033 per cent. At this concentration both aminopterin and folic acid had an inhibiting effect on the tyrosine-tyrosinase reaction ; the results are shown in Table II.

TABLE II.—*The Oxidation of Tyrosine to Melanin in the Presence of* 0·033 *per cent of Aminopterin and Folic Acid.*

Increments of "E".

| Time in minutes. | Tyrosine. | Tyrosine with aminopterin. | Tyrosine with folic acid. |
|---|---|---|---|
| 60 | ·0165 | ·0148 | ·0119 |
| 120 | ·0386 | ·0301 | ·0265 |
| 180 | ·0514 | ·0433 | ·0391 |
| 240 | ·0646 | ·0569 | ·0468 |
| 300 | ·0755 | ·0653 | ·0574 |
| 360 | ·0838 | ·0710 | ·0654 |

It will be noted that the inhibition by folic acid is greater than that by aminopterin. These results were confirmed by two repetitions.

The effect of aminopterin and folic acid on the autoxidation of dopa was then investigated. (Dopa is the first intermediate product in the autoxidation of tyrosine to melanin. Tyrosinase is only necessary for the addition of the second hydroxyl to the benzene ring. After this has been done the oxidation will proceed without the aid of the enzyme (Raper, 1926).)

Concentrations of 0·0166 per cent of each of these substances were first used, the concentration of dopa being 0·005 per cent. Both substances produced an acceleration of the reaction, the effect of the aminopterin being much greater than that of folic acid. In succeeding experiments the concentration of aminopterin was reduced until a level of 0·00005 per cent was reached, at which level the accelerating effect was still evident (Table III).

TABLE III.—*The Effect of* 0·00005 *per cent Aminopterin on the Autoxidation of Dopa.*

Increments of "E".

| Time in minutes. | Dopa. | Dopa plus aminopterin. |
|---|---|---|
| 60 | ·0317 | ·0293 |
| 120 | ·0893 | ·0900 |
| 180 | ·1621 | ·1685 |
| 240 | ·2306 | ·2458 |
| 300 | ·2945 | ·3166 |

Finally a concentration of 0·0000125 per cent of aminopterin was used (approximately 1 part in 8,000,000), and at this level the accelerating effect was so small that it could not be distinguished from the probable experimental errors. This experiment was therefore made 17 times in all. On 15 occasions there was acceleration averaging approximately 0·3 per cent as compared with the control. On the assumption that the errors inevitably present will tend to operate equally in either direction, the probability of obtaining 15 results out of 17 in one direction is (approximately) only 1 in 500, so that the results are statistically significant.

The possibility of the acceleration being due to change in pH was reconsidered. The pH of the various solutions employed was estimated, electrometrically, using a glass electrode. The values found are given in Table IV.

TABLE IV.—*pH Values of Solutions.*

| Solution. | pH. |
|---|---|
| Buffer | 7·36 |
| Dopa 0·005 % | 7·28 |
| Dopa 0·005% plus aminopterin 0·0125% | 7·26 |
| Dopa 0·005% plus aminopterin 0·025% | 7·23 |
| Dopa 0·005% plus folic acid 0·0125% | 7·25 |
| Dopa 0·005% plus folic acid 0·025% | 7·23 |

There is a slight shift to the acid side of the original pH of the buffer solution. The autoxidation of dopa buffered at pH 7·2, 7·4 and 7·6 was therefore investigated. As anticipated, the reaction proceeded more slowly at the more acid pH (Table V).

TABLE V.—*The Effect of pH on the Autoxidation of Dopa.*

| Time in minutes. | Increments of " E ". pH 7·2. | pH 7·4. | pH 7·6. |
|---|---|---|---|
| 60 | ·0134 | ·0179 | ·0271 |
| 120 | ·0465 | ·0586 | ·0871 |
| 180 | ·0876 | ·1091 | ·1631 |

It is evident that the accelerating effect of aminopterin and folic acid is not due to change in pH. What little change there is operates *against* such an acceleration.

### DISCUSSION.

There is now evidence that three substances used in the treatment of leukaemia —urethane, nitrogen mustard and aminopterin—have an accelerating action on the autoxidation of dopa. Urethane and aminopterin have an inhibiting effect on the enzymatic oxidation of tyrosine, but only at concentrations much greater than those producing the acceleration of the autoxidation. The nitrogen mustard produced no inhibition of the enzymatic reaction even at a concentration of 0·2 per cent. Also folic acid produces this acceleration of the autoxidation. It might be supposed from this that any substances inhibiting the tyrosine-tyrosinase reaction would also accelerate the autoxidation of dopa. That this is not so is shown by the action of hydroquinone mono-benzyl ether, which inhibits both of these oxidations (Lea, 1951). These findings may have a bearing on the probable nature of the maturation factor in normal leucopoiesis.

Apparently the earliest work pointing to the presence of such a factor was that of Sabin, Cunningham, Doan and Kindwall in 1925. Since then it has been suggested that folic acid (Wilson, Doan, Saslaw and Schwab, 1942) and pyridoxine (Cantor and Scott, 1945) may be this factor. Dacie, Dresner, Mollin and White (1950) reported that after treatment of acute leukaemia by aminopterin there appeared to be a return to normal haemopoiesis. It is now suggested that this factor, whatever its nature, acts by promoting autoxidative processes in the normal production of leucocytes. Such a suggestion allows the inclusion of folic acid with substances recognized as therapeutic agents in the leukaemias. It is to be noted that folic acid ceased to produce an accelerating effect on the autoxidation of dopa at a concentration of approximately 1 part in 100,000, whereas the effect of aminopterin was still obvious at a level of 1 part in 2,000,000, and detectable by statistical methods at a level of 1 part in 8,000,000.

It appeared that still further evidence bearing on this suggestion might be obtained by comparing the degrees of acceleration of the autoxidation of dopa produced by urethane, nitrogen mustard and aminopterin. This was done, and it was found that the accelerating effects of these substances were in the ratio of urethane 1, nitrogen mustard 100, aminopterin 1000. These figures are approximate, and indicate only the order of the differences. It is of interest to compare these orders of magnitude of acceleration with the amounts of these substances used therapeutically, and with their clinical effects.

It must be emphasized that there is no suggestion that the autoxidation of dopa plays any part in either normal or abnormal leucopoiesis ; or that any one of the three substances so far investigated is the actual maturation factor. All that it is intended to convey here is that the maturation factor may be concerned with some form of autoxidative process, and that leukaemia may be a result of its dysfunction. These suggestions are put forward only as a hypothesis for investigation, with the further suggestion that it might be of interest, and possibly of advantage, to investigate the effects on the reticuloses of other substances which accelerate autoxidations. There appears to be no association between these findings and the release factor of normal, or abnormal, leucopoiesis.

### SUMMARY.

(1) Aminopterin and folic acid inhibit the tyrosine-tyrosinase reaction, the effect of folic acid being greater than that of aminopterin.

(2) Aminopterin and folic acid accelerate the autoxidation of dopa, the effect of aminopterin being much greater than that of folic acid.

(3) It is suggested that these results, taken in conjunction with similar findings for urethane and di-(2-chloroethyl)-methylamine hydrochloride, admit of the hypothesis that the maturation factor in leucopoiesis is concerned with the promotion of autoxidations.

(4) It is suggested that the study of other substances having this accelerating effect on autoxidations might help in the solution of the problem of the leukaemias.

The work has been carried out with the aid of a grant from the Government Grant Committee of the Royal Society. I am greatly indebted to the Lederle Laboratories Division of Cyanamid Products Ltd. for generous supplies of aminopterin and folic acid.

### REFERENCES.

Cantor, M. M., and Scott, J. W.—(1945) *Canad. med. Ass. J.*, **52**, 368.

Dacie, J. V., Dresner, E., Mollin, D. L., and White, J. C.—(1950) *Brit. med. J.*, i, 1447.

Lea, A. J.—(1950) *Brit. J. Cancer*, **4**, 341.—(1951) *Nature*, **167**, 906.

Raper, H. S.—(1926) *Biochem. J.*, **20**, 735.

Sabin, F. R., Cunningham, R. S., Doan, C. A., and Kindwall, J. A.—(1925) *Johns Hopk. Hosp. Bull.*, **37**, 14.

Wilson, H. E., Doan, C. A., Saslaw, S., and Schwab, J. L.—(1942) *Proc. Soc. exp. Biol., N.Y*,, **50**, 341.